# Developing a predictive model for lower extremity deep vein thrombosis in acute ischemic stroke using a nomogram

**DOI:** 10.3389/fneur.2025.1506959

**Published:** 2025-06-16

**Authors:** Weicong Chen, Chaohua Cui, Changsheng Lai

**Affiliations:** ^1^Life Science and Clinical Medicine Research Center, Affiliated Hospital of Youjiang Medical University for Nationalities, Baise, China; ^2^Clinical College of Youjiang Medical University for Nationalities, Baise, China; ^3^Red Cross Hospital of Yulin City, Yulin, China

**Keywords:** acute ischemic stroke, deep vein thrombosis, risk factors, prediction, nomogram

## Abstract

**Background:**

Deep vein thrombosis (DVT) is a prevalent complication among patients with acute ischemic stroke (AIS). However, there remains a deficiency of patient-specific predictive models. This study aims to develop a nomogram to estimate the risk of lower extremity DVT in AIS patients during the acute phase (within 14 days of onset).

**Methods:**

This retrospective multicenter study analyzed 391 eligible AIS patients from two tertiary hospitals in Guangxi, China. Sixty-three clinical variables encompassing demographic profiles, clinical characteristics, laboratory parameters, and therapeutic interventions were systematically extracted from electronic health records. All participants underwent standardized Doppler ultrasound assessments for bilateral lower extremity DVT within 14 days of symptom onset. Variable selection via backward stepwise logistic regression informed nomogram construction, with model performance evaluated through calibration curves and decision curve analysis.

**Results:**

Data from one hospital were used as the modeling cohort, while data from another hospital were used for external validation. Multivariate logistic regression analysis showed that gender, age, diabetes, anemia, bed rest exceeding 3 days, and medium-frequency electrical therapy are independent risk factors for DVT in AIS patients. A nomogram was developed based on these six independent risk factors, with the area under the ROC curve (AUC) for predicting DVT risk within 14 days post-AIS being 0.812 for the modeling cohort and 0.796 for the external validation, indicating good predictive performance. Calibration of the nomogram showed Hosmer-Lemeshow test results with *p* values of 0.200 for the modeling set and 0.432 for the validation set, indicating good model consistency. In decision curve analysis, the nomogram demonstrated superior net benefit over staging systems across a wide range of threshold probabilities.

**Conclusion:**

We developed a nomogram to personalize the prediction of DVT risk in patients with AIS, assisting healthcare professionals in the early identification of high-risk groups for DVT and in implementing appropriate interventions to effectively prevent its occurrence.

## Introduction

Stroke ranks as the second most common cause of death worldwide, characterized by high incidence, elevated disability rates, and significant recurrence (1–3). Stroke typically manifests with clinical features including paralysis and mobility impairment, and without standardized preventive measures, complications such as venous thromboembolism (VTE) are likely (4, 5). VTE comprises deep vein thrombosis (DVT) and pulmonary embolism (PE). Migration of lower limb DVT clots often precipitates PE, representing approximately 25% of early post-stroke mortality (5, 6). DVT risk peaks during the initial two-week period post-stroke, with possible onset as early as day two and peaking between days two and seven (7, 8). Ischemic stroke accounts for the majority of stroke cases, representing approximately 80% of total stroke incidence (5). Studies report DVT incidence ranging from 18.0 to 23.5% in acute ischemic stroke (AIS) patients (9, 10). DVT development in AIS patients impedes rehabilitation progress, prolongs hospitalization, and increases disability and mortality rates (11, 12).

DVT following stroke frequently manifests asymptomatically in clinical practice. Cognitive, speech, or consciousness impairments may further compromise symptom reporting, leading to diagnostic omissions (13, 14). The clinical dilemma persists in balancing pharmacological thromboprophylaxis against bleeding risks in ischemic stroke management (6, 15). Current diagnostic gold standards (Doppler ultrasound and venography) demonstrate limited predictive utility and time-dependent sensitivity (16). Currently, commonly utilized tools in clinical practice for assessing VTE risk, such as the Widely used VTE risk assessment tools (Caprini RAM, Padua Score) exhibit inadequate specificity for stroke populations. Despite multifactorial DVT etiology, evidence remains limited regarding AIS-specific predictors, with no validated algorithms for identifying high-risk patients.

The nomogram, a graphical predictive instrument integrating multiple risk factors, has demonstrated clinical utility across various diseases (17, 18). This investigation comprehensively evaluates demographic, clinical, laboratory, and therapeutic parameters to develop a personalized nomogram for acute-phase DVT prediction (≤14 days post-AIS onset), aiming to enhance clinical decision-making and preventive strategies.

## Materials and methods

### Study design and participants

This retrospective observational study comprised two cohorts: 310 AIS inpatients meeting inclusion criteria from the Affiliated Hospital of Youjiang Medical University for Nationalities in Guangxi, China (January 2022–January 2024) formed the modeling set, while 81 AIS inpatients from Baidong Hospital in Baise City (January 2024–January 2025) constituted the external validation cohort. All patients were required to meet the following inclusion criteria: (1) Age ≥ 18 years. (2) Diagnosed with AIS according to the International Classification of Diseases Tenth Revision (ICD-10) code I63, as confirmed by cranial CT or MRI. (3) The initial onset of AIS occurred within 72 h from the onset to admission. A bilateral lower extremity venous Doppler ultrasound examination was completed within 14 days of onset. Patients who met any exclusion criteria were excluded, which included: (1) History of VTE. (2) History of prior stroke, cranial trauma, or organic brain lesions. (3) History of coagulation disorders or hematological conditions. (4) History of lower limb fractures, trauma, or other factors resulting in limb dysfunction. (5) History of active tumors occurring within 3 months prior to onset. (6) Medical records are severely lacking. Ultimately, 391 patients with AIS met the inclusion criteria for this study.

### Date collection

Demographic characteristics comprised age and gender. Clinical feature parameters comprised smoking history, alcohol consumption history, lacunar infarction, large cerebral infarction, fever, atrial fibrillation, coronary heart disease, hypertension, diabetes, anemia, disturbances of consciousness, National Institute of Health stroke scale (NIHSS), Modified Rankin Scale (mRS), the Watanabe water drinking test (WDT), lower limb muscle strength, and the Barthel Index. laboratory parameters included leukocytes, neutrophils, lymphocytes, monocytes, hematocrit, platelets, mean platelet volume, systemic immune-inflammation index (SII), systemic inflammatory response index (SIRI), neutrophil/lymphocyte ratio (NLR), platelet/lymphocyte ratio (PLR), lymphocyte/monocyte ratio (LMR), mean platelet volume/lymphocyte ratio (MPVLR), albumin, total cholesterol, triglycerides, high-density lipoprotein, low-density lipoprotein, lipoprotein (a), homocysteine, serum creatinine, blood urea nitrogen, creatinine/urea nitrogen ratio, uric acid, lactate dehydrogenase, prothrombin time, international normalized ratio, activated partial thromboplastin time, thrombin time, and fibrinogen levels. All laboratory parameters for the patients were conducted within 24 h of admission. Clinical treatment parameters encompassed the use of antibiotics, subclavian deep vein catheterization, antiplatelet therapy, intermittent pneumatic compression (IPC) therapy, anticoagulant treatment, ventilator-assisted respiration, diuretic therapy, medium-frequency electrotherapy, acupuncture treatment, movement therapy, rehabilitation therapy, characteristics of cerebral infarction lesions, bed rest duration exceeding 3 days, digital subtraction angiography, and vascular open surgery.

### Data processing

If 10% of the data for a specific research factor is missing, that factor will be excluded; similarly, if 10% of the data for a particular patient is missing, that patient will be removed from the analysis. For quantitative data, the mean imputation method will be employed to address missing values. For categorical variables, the mode imputation method will be applied to address missing values. Duplicate records and outliers will be eliminated. By processing the data according to these methods, we aim to minimize bias arising from missing or anomalous data, thereby enhancing the accuracy of the experimental results.

### Definition of DVT

All AIS patients received comprehensive Doppler ultrasound evaluation within 14 days post-onset to detect DVT in lower extremities. Diagnostic criteria included visualization of filling defects, blood flow absence, heterogeneous intraluminal echoes, or venous non-compressibility during pressure application. Radiologists conducted all imaging procedures and interpretations. Systematic examination protocols were implemented: Deep veins in thigh, popliteal fossa, and calf regions were transversely assessed at 2 cm intervals. Proximal evaluation extended from the inguinal ligament to adductor canal levels in supine position. The popliteal vein was specifically examined at the upper calf trifurcation, with thrombi detected at or above this anatomical landmark classified as proximal DVT. Subsequent evaluation of remaining calf veins to ankle level identified distal DVT cases. Diagnostic confirmation occurred irrespective of thrombus location, multiplicity, or temporal development characteristics.

### Statistical analysis

Participants were categorized into two groups: those with DVT and those without. The data were evaluated for normal distribution using the Kolmogorov–Smirnov test. Continuous variables are presented as means ± standard deviation (SD) or medians (P_25_, P_75_). Categorical variables are reported as frequencies (*n*) and percentages (%). For numerical variables, differences between the two groups were analyzed using Student’s t-test (for parametric data) or the Mann–Whitney U test (for non-parametric data). The associations between each independent variable and distal DVT outcomes were examined using Pearson’s χ^2^ test and Fisher’s exact test, respectively. To further identify independent predictors of DVT, we conducted a multivariable logistic regression analysis employing a backward stepwise method to determine the final multivariable model for each primary outcome. Factors were excluded based on a likelihood ratio test with a significance level of *p* < 0.05. A personalized nomogram prediction model was developed based on the regression coefficients of the final variables. The model’s predictive ability was quantified using the area under the receiver operating characteristic curve (AUC). Furthermore, the model’s predictive performance was validated using the Hosmer-Lemeshow test. A Hosmer-Lemeshow test *p*-value greater than 0.05 indicates no significant difference between observed and predicted probabilities, suggesting good calibration. To determine clinical value, decision curve analysis (DCA) was implemented to quantify the nomogram’s net benefit across multiple threshold probabilities, providing robust evidence for its practical utility in clinical decision-making. Statistical analyses were performed using SPSS software version 26.0 (IBM, United States) and R software version 4.3.0.

## Results

### Patient characteristics

This study comprised 391 patients, divided into a modeling cohort of 310 and an external validation cohort of 81. In the modeling cohort, 70.3% (218) were male and 29.7% (92) were female, with a mean age of 61.3 ± 11.8 years, ranging from 28 to 87 years. The incidence of lower extremity DVT was 24.8% (77 patients). The external validation cohort included 48 males (59.3%) and 33 females (40.7%), with a mean age of 61.7 ± 10.86 years, ranging from 40 to 86 years, and a lower extremity DVT incidence of 24.7% (20 patients). Statistical analysis found no significant differences in baseline characteristics between the cohorts.

### Univariate analysis of DVT risk factors in AIS patients

Table 1 displays the univariate analysis results of factors associated with DVT among AIS patients in the modeling cohort. The analysis reveals significant differences among the groups in nine risk factors: gender, age, smoking history, alcohol consumption history, diabetes, anemia, hematocrit levels, medium-frequency electrotherapy and bed rest duration exceeding 3 days (all *p* values < 0.05).

**Table 1:** Baseline data of AIS patients with and without DVT in the training set.

Characteristics	No DVT (*n* = 233)	DVT (*n* = 77)	Total (*n* = 310)	*p*-value
Demographic characteristics				
Age (years)	59.07 ± 11.41	68.14 ± 10.33	61.32 ± 11.79	<0.001
Gender				<0.001
Male	180 (77.3%)	38 (49.4%)	218 (70.3%)	
Female	53 (22.7%)	39 (50.6%)	92 (29.7%)	
Clinical feature parameters				
Smoking history	91 (39.1%)	11 (14.3%)	102 (32.9%)	<0.001
Alcohol consumption history	92 (39.5%)	13 (16.9%)	105 (33.9%)	<0.001
Lacunar infarction	109 (46.8%)	35 (45.5%)	144 (46.5%)	0.840
Large cerebral infarction	23 (9.9%)	5 (6.5%)	28 (9.0%)	0.370
Fever	63 (27.0%)	26 (33.8%)	89 (28.7%)	0.258
Atrial fibrillation	8 (3.4%)	7 (9.1%)	15 (4.8%)	0.089
Coronary heart disease	8 (3.4%)	4 (5.2%)	12 (3.9%)	0.723
Hypertension	177 (76%)	56 (72.7%)	233 (75.2%)	0.569
Diabetes	32 (13.7%)	19 (24.7%)	51 (16.5%)	0.025
Anemia	56 (24.0%)	39 (50.6%)	95 (30.6%)	<0.001
Disturbances of consciousness	39 (16.7%)	12 (15.6%)	51 (16.5%)	0.813
NIHSS	6 (3,10.5)	7 (3,13)	7 (3,11)	0.337
mRS	4 (2,4)	4 (2,4)	4 (2,4)	0.404
WDT	2 (1,3)	2 (1,3)	2 (1,3)	0.506
Lower limb muscle strength	3 (2,4)	3 (2,4)	3 (2,4)	0.278
Barthel Index	30 (15,55)	30 (11.25,45)	30 (15,55)	0.248
Laboratory parameters				
Leukocytes	9.49 ± 2.92	9.51 ± 2.99	9.49 ± 2.93	0.960
Neutrophils	7.94 ± 2.94	7.27 ± 3.06	6.70 (4.81,9.17)	0.778
Lymphocytes	1.54 (1.16,2.08)	1.45 (1.09,2.05)	1.51 (1.12,2.07)	0.281
Monocytes	0.59 ± 0.25	0.60 ± 0.28	0.58 (0.41,0.74)	0.966
Hematocrit	44.1 (41.25,47.5)	41.3 (37.45,44)	43.3 (40.28,46.95)	<0.001
Platelets	263.6 ± 72.7	247.5 (207.5,300.25)	257 (213.5,304.25)	0.379
Mean platelet volume	10.2 (9.5,11)	10.36 (9.7,11.3)	10.48 ± 1.12	0.327
SII	941.67 (653.38,1924.86)	1115.06 (640.43,2087.77)	979.74 (652.22,1941.54)	0.536
SIRI	2.26 (1.36,3.76)	2.38 (1.35,4.55)	2.89 (1.36,3.86)	0.651
NLR	3.85 (2.55,6.90)	4.14 (2.61,7.62)	3.92 (2.56,7.18)	0.354
PLR	164.06 (114.63,229.81)	174.79 (115.16,259.18)	165.66 (114.80,236.33)	0.494
LMR	2.79 (1.99,3.89)	2.71 (2.10,4.09)	2.78 (2,3.91)	0.662
MPVLR	6.71 (4.88,9.)	7.11 (5.03,9.89)	6.79 (4.97,9.21)	0.218
Albumin	41.7 (38.9,45)	40.75 (38.53,43.38)	41.4 (38.8,44.6)	0.057
Total cholesterol	4.69 ± 1.15	4.55 ± 1.18	4.66 ± 1.16	0.435
Triglycerides	1.31 (0.97,2.12)	1.24 (0.90,1.655)	1.30 (0.95,1.85)	0.159
High-density Lipoprotein	1.14 (0.96,1.68)	1.17 (0.96,1.51)	1.15 (0.96,1.39)	0.620
Low-density Lipoprotein	3.25 ± 1.11	3.19 ± 1.15	3.11 (2.48,3.91)	0.812
Lipoprotein (a)	30.7 (15.4,65.65)	27.4 (14.33,79.36)	29.75 (14.95,66.82)	0.656
Homocysteine	12.8 (10.3,16.15)	13.1 (9.63,16.4)	12.85 (10.2,16.12)	0.694
Serum creatinine	78 (69,92.5)	81 (66.25,100)	79 (69,94)	0.638
Blood urea nitrogen	4.8 (3.8,5.8)	5.05 (3.92,6.68)	4.8 (3.8,5.93)	0.089
Cr/BUN	17.65 ± 5.46	17.05 ± 6.32	17.16 (13.08,21.15)	0.211
Uric acid	360 (282,429)	352.29 ± 101.05	358 (278.75,430)	0.384
Lactate dehydrogenase	192 (163,224.5)	195 (169.5,231)	192 (165,225.5)	0.468
PT	11.1 (10.6,11.9)	11.4 (10.9,11.8)	11.2 (10.68,11.9)	0.099
INR	0.95 (0.9,1.02)	0.98 (0.92,1.03)	0.96 (0.91,1.02)	0.057
APTT	26.2 (23.4,29.65)	26.1 (24.03,28.7)	26.2 (23.4,29.4)	0.866
TT	17.7 (16.5,19.1)	17.65 (16.7,18.68)	17.65 (16.6,19)	0.486
FIB	2.97 (2.41,3.44)	2.97 (2.54,3.65)	2.97 (2.42,3.44)	0.522
Clinical treatment parameters				
Use of antibiotics	53 (22.7%)	21 (27.3%)	74 (23.9%)	0.419
Subclavian deep vein catheterization	33 (14.2%)	10 (13.0%)	43 (13.9%)	0.796
Antiplatelet therapy				0.792
None	4 (1.7%)	2 (2.6%)	6 (1.9%)	
Single	122 (52.4%)	39 (50.6%)	161 (51.9%)	
Dual linkage	107 (45.9%)	36 (46.8%)	143 (46.1%)	
IPC	221 (94.8%)	70 (90.9%)	291 (93.9%)	0.270
Anticoagulant treatment	4 (1.7%)	1 (1.3%)	5 (1.6%)	1.000
Ventilator-assisted respiration	14 (6%)	4 (5.2%)	18 (5.8%)	1.000
Diuretic therapy	104 (44.6%)	41 (53.2%)	46.8 (145%)	0.189
Medium-frequency electrotherapy	165 (70.8%)	44 (57.1%)	209 (67.4%)	0.026
Acupuncture treatment	135 (57.9%)	37 (48.1%)	172 (55.5%)	0.130
Movement therapy	161 (69.1%)	46 (59.7%)	207 (66.8%)	0.131
Rehabilitation therapy	172 (73.8%)	54 (70.1%)	226 (72.9%)	0.528
Characteristics of cerebral infarction lesions				0.828
Unilateral brain	91 (39.1%)	29 (37.7%)	120 (38.7%)	
Bilateral brain	142 (60.9%)	48 (62.3%)	190 (61.3%)	
Bed rest duration exceeding 3 days	92 (39.5%)	54 (70.1%)	146 (47.1%)	<0.001
DSA	177 (76%)	51 (66.2%)	228 (73.5%)	0.093
Vascular open surgery				0.962
None	135 (57.9%)	46 (59.7%)	181 (58.4%)	
Intravenous thrombolysis	19 (8.2%)	6 (7.8%)	25 (8.1%)	
Interventional therapy	79 (33.9%)	25 (32.5%)	104 (33.5%)	

NIHSS, National Institute of Health stroke scale; mRS, Modified Rankin Scale; WDT, the Watanabe water drinking test; SII, systemic immune-inflammation index, SIRI, systemic inflammatory response index, NLR, neutrophil/lymphocyte ratio, PLR, platelet/lymphocyte ratio, LMR, lymphocyte/monocyte ratio, MPVLR, mean platelet volume/lymphocyte ratio, Cr/BUN, creatinine/urea nitrogen ratio; IPC, intermittent pneumatic compression, DSA, digital subtraction angiography.

### Multivariable analysis of DVT risk factors in Als patients

The multivariable model identifies several factors associated with an increased risk of DVT in AIS patients, including female gender (OR = 2.214, 95% CI: 1.190–4.122), age (OR = 1.060, 95% CI: 1.030–1.091), anemia (OR = 2.231, 95% CI: 1.191–4.180), diabetes (OR = 3.318, 95% CI: 1.551–7.099), and bed rest duration exceeding 3 days (OR = 2.858, 95% CI: 1.514–5.394). Medium-frequency electrotherapy (OR = 0.406, 95% CI: 0.215–0.765) is identified as a factor associated with a reduced risk of DVT (Table 2).

**Table 2:** Multivariate logistic regression analysis with respect to DVT in AIS patients.

Characteristics	*p*-value	OR (95% CI)
Gender	0.012	2.214 (1.190–4.122)
Age	<0.001	1.060 (1.030–1.091)
Anemia	0.012	2.231 (1.191–4.180)
Diabetes	0.002	3.318 (1.551–7.099)
Bed rest duration exceeding 3 days	0.001	2.858 (1.514–5.394)
Medium-frequency electrotherapy	0.005	0.406 (0.215–0.765)

### Performance and validation of the nomogram

**Figure 1:**
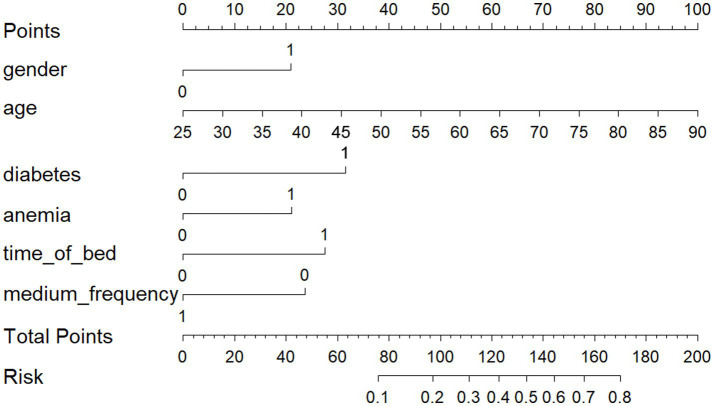
A nomogram predicting the presence of deep vein thrombosis (DVT) in AIS patients based on gender, age, anemia, diabetes, Bed rest duration exceeding 3 days, and medium-frequency electrotherapy. Regarding gender, 0 represents male and 1 represents female. For diabetes, anemia, and medium-frequency electrotherapy, 0 indicates absence, while 1 indicates presence. For bed rest duration, 0 indicates a duration of 3 days or fewer, while 1 indicates a duration exceeding 3 days.

**Figure 2:**
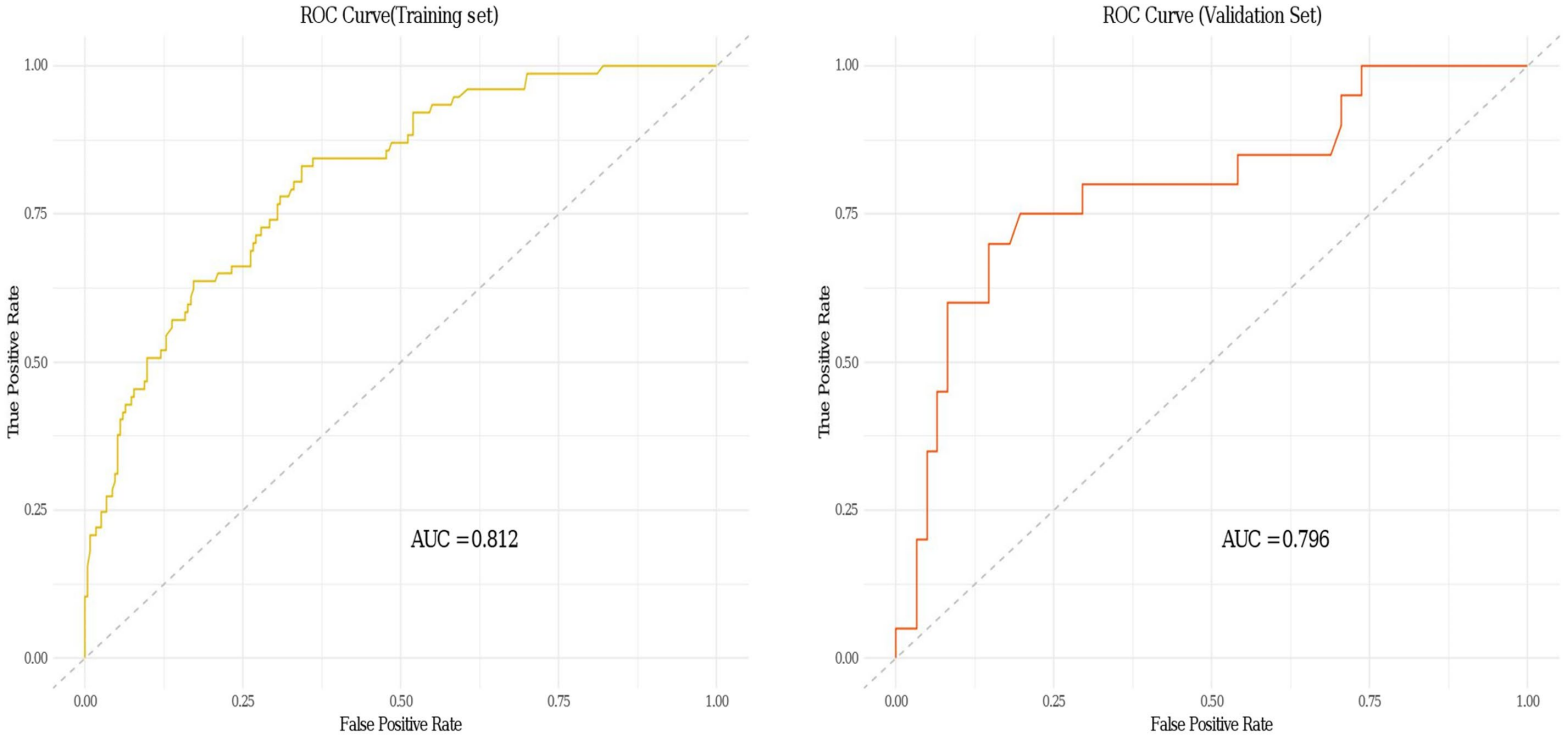
Receiver operating characteristic (ROC) curves of the clinical prediction model for deep vein thrombosis (DVT) in acute ischemic stroke (AIS) patients in both the training and validation sets.

**Figure 3:**
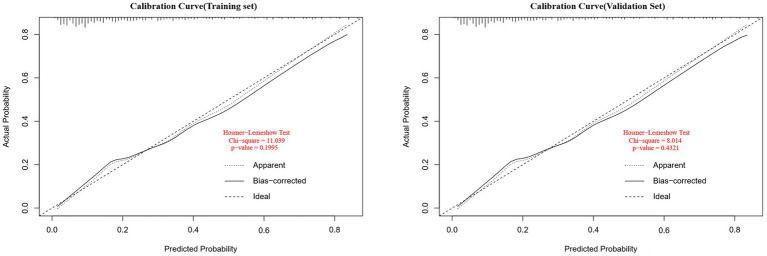
Calibration curves for the deep vein thrombosis (DVT) nomogram prediction in the training and validation sets. The x-axis represents the predicted DVT risk. The y-axis represents the actual diagnosed DVT. The diagonal dotted line depicts a perfect prediction by an ideal model. The solid line reflects the performance of the nomogram; a closer fit to the diagonal dotted line indicates a better prediction (Hosmer-Lemeshow test, *p* > 0.05, indicating goodness of fit).

**Figure 4:**
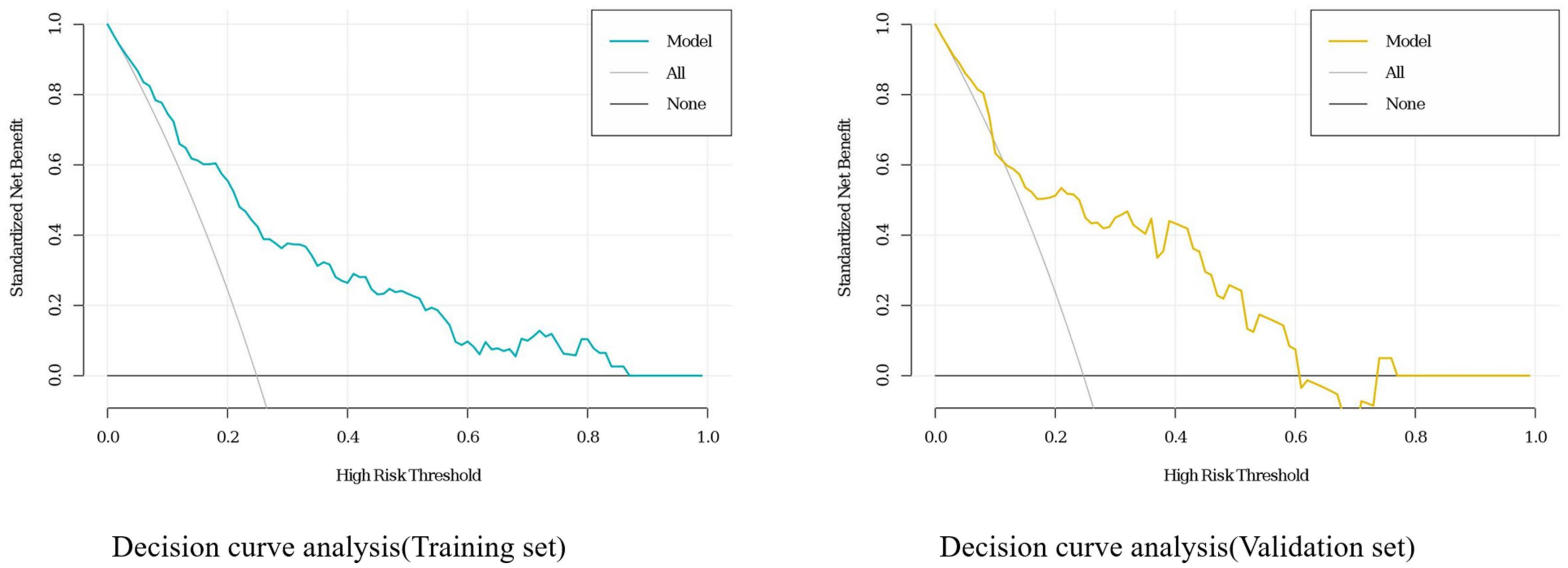
Decision curve analysis (DCA) for the deep vein thrombosis (DVT) nomogram in the training and validation sets of acute ischemic stroke (AIS) patients.

A personalized nomogram prediction model was developed based on six independent predictive factors: gender, age, diabetes, anemia, medium-frequency electrical therapy, and bed rest exceeding 3 days (Figure 1). Table 3 shows the univariate analysis results of DVT-related factors in AIS patients in the external validation cohort. We performed external validation of the nomogram’s model performance, with AUCs of 0.812 and 0.796 in the modeling and validation cohorts, respectively, indicating good predictive efficacy (Figure 2). The calibration of the nomogram showed Hosmer-Lemeshow test results with *p* values of 0.200 in the modeling set and 0.432 in the validation set, indicating good model consistency (Figure 3). In decision curve analysis, the nomogram showed superior net benefit over staging systems across a wide range of threshold probabilities (Figure 4).

**Table 3:** Baseline data of AIS patients with and without DVT in the validation set.

Characteristics	No DVT (*n* = 61)	DVT (*n* = 20)	Total (*n* = 81)	*p*-value
Age (years)	58.98 ± 10.42	70.00 ± 7.56	61.7 ± 10.86	<0.001
Gender				0.655
Male	37 (45.7%)	11 (13.6%)	48 (59.3%)	
Female	24 (29.6%)	9 (11.1%)	33 (40.7%)	
Diabetes	11 (13.6%)	4 (4.9%)	15 (18.5%)	0.844
Anemia	14 (17.3%)	9 (11.1%)	23 (28.4%)	1.000
Medium-frequency electrotherapy	35 (43.2%)	12 (14.8%)	47 (58.0%)	0.837
Bed rest duration exceeding 3 days	16 (19.8%)	14 (17.3%)	30 (37.0%)	<0.001

## Discussion

This study identified six easily obtainable and assessable predictive variables: age, gender, anemia, diabetes, bed rest duration exceeding 3 days, and medium-frequency electrotherapy. A personalized prediction nomogram for DVT in AIS patients during the acute phase was developed. This tool assists healthcare professionals in predicting the occurrence of DVT in AIS patients in the acute phase. The model demonstrated strong performance in predicting the risk of DVT in AIS patients during the acute phase. External validation of the model demonstrated good discrimination and calibration performance, along with consistency in interval testing. This indicates that the nomogram can be applied widely and accurately in large sample analyses.

The primary mechanisms underlying the occurrence of DVT include vascular wall injury, hypercoagulability, and slow blood flow. Our model is founded on these three elements. It is believed that DVT is typically influenced by multiple risk factors. These risk factors often change dynamically, presenting a significant challenge in predicting DVT. When multiple risk factors combine to exceed the “thrombus formation threshold,” they may lead to the occurrence of DVT within a specific time frame. For instance, a particular risk factor for DVT may suggest a high risk; however, if it does not combine with other risk factors to exceed the “thrombus formation threshold,” it ultimately does not lead to the occurrence of DVT. Conversely, the reverse is also true. Therefore, it is essential to closely monitor the dynamic changes in DVT risk factors to promptly identify high-risk factors for early intervention, thereby effectively reducing the incidence of DVT.

Consistent with previous research, this study identifies advanced age as an independent risk factor for DVT in AIS patients, indicating that older individuals are more susceptible to DVT (11, 19). Advanced age is a well-established independent risk factor (20, 21). Cai et al. (11) conducted a large-scale, multi-center study and found that for every 10-year increase in age among AIS patients, the risk of developing DVT increases by 1.108 times. A meta-analysis indicated that age is one of the most frequently utilized predictive factors in risk prediction models for DVT in acute stroke patients (22). Numerous predictive models identify age as a risk factor for DVT following a stroke. In older patients, particularly among the elderly, factors such as declining mobility, slow venous return in the lower limbs, decreased vascular elasticity, increased susceptibility of vascular walls to injury, weakened calf muscle pump function, and elevated levels of reactive oxygen species (ROS) may exacerbate DVT (11, 23). Wang et al. (24) found that levels of coagulation factors increase with age, and higher levels of factors VIII, IX, and XI are positively correlated with the risk of venous thrombosis.

Consistent with previous research, this study identifies female sex as a significant risk factor for DVT (6, 25). The underlying reasons for the increased risk in women remain unclear and may be associated with factors such as hormonal fluctuations, hormone replacement therapy, pregnancy or the postpartum period, and a history of uterine surgery. These factors can contribute to endothelial dysfunction, a hypercoagulable state, and blood stasis (26–28). Estrogen, as a means of managing menopausal syndrome in women, studies have shown that while it alleviates symptoms, it carries significant potential risks, with the incidence of VTE possibly increasing by 2–4 times (29). Current research has elucidated its multiple pathways of action: One mechanism is through activating endothelial cell function and enhancing platelet activity, thus increasing the concentration of von Willebrand factor; another pathway is the promotion of liver synthesis of coagulation factors such as FVII, FVIII, and fibrinogen, accelerating thrombin generation and fibrin network construction; furthermore, this hormone also inhibits the expression of natural anticoagulants such as tissue factor pathway inhibitor (30).

The mechanisms linking anemia and DVT remain unclear. In a large-scale study, Zakai et al. (31) developed a VTE risk assessment model and identified low hemoglobin levels as an independent risk factor (OR = 1.47, 95% CI: 1.05–2.09). A study involving 6,861 patients demonstrated that low hemoglobin levels are associated with an elevated risk of symptomatic DVT (RR = 2.29, 95% CI: 1.12–4.68, *p* = 0.019). After adjusting for confounding factors, low hemoglobin levels continued to be associated with an elevated likelihood of VTE (adjusted OR = 1.71, 95% CI: 1.09–2.69, *p* = 0.020). It is suggested that potential risk factors for venous thromboembolism, including nutritional deficiencies, chronic inflammation, cancer and its associated chemotherapy, as well as acute illnesses, may contribute to anemia, thereby increasing the risk of VTE (32). Furthermore, Zhang et al. (33) found that anemia is an independent risk factor for DVT, suggesting that this may be related to several mechanisms: anemia stimulates platelet production, increases plasminogen activator inhibitors, and reduces fibrinolytic activity, leading to increased stress on the vascular endothelium, resulting in endothelial injury and a heightened risk of thrombosis. A study assessing the predictive role of complete blood count parameters for DVT found that hemoglobin is an independent predictor of DVT, with an AUC of 0.765 (95% CI: 0.683–0.848, *p* < 0.001). This suggests that the mechanisms by which anemia contributes to DVT are similar to those previously mentioned (34).

This study identifies diabetes as an independent risk factor for DVT in patients with AIS. A large cohort study conducted in a Taiwanese population reported that the risk of DVT in diabetic patients was 1.43 times higher than that in the control cohort (adjusted hazard ratio [aHR] = 1.43, 95% CI: 1.23–1.65) (35). Another large retrospective case–control study involving an Austrian cohort reported that the risk of VTE in patients with type 2 diabetes was 1.4 times higher than that in the control cohort (OR = 1.4, 95% CI: 1.36–1.43) (36). Both studies suggest that diabetes is an independent risk factor for VTE. The study conducted by Kaur et al. (37) suggests that the metabolic environment characterized by glycemia and insulin resistance in type 2 diabetes may contribute to endothelial dysfunction, platelet hyperactivity, oxidative stress, and low-grade inflammation, which adversely affect the vascular wall and enhance vasoconstriction, thereby promoting thrombosis. Two meta-analyses suggest that diabetes is an independent risk factor for VTE, with the first reporting an OR of 1.42 (95% CI: 1.12–1.77) (38), and the second reporting a hazard ratio (HR) of 1.35 (95% CI: 1.17–1.55) (39). This study observed differences in the risk of DVT among diabetic patients in comparison to studies conducted in Western countries, which may be related to regional and ethnic variations.

This study identifies bed rest duration exceeding 3 days as an independent risk factor for DVT in patients with AIS. Two clinical reviews indicate that bed rest is a significant risk factor for DVT. The Caprini Risk Assessment Model, frequently utilized for surgical inpatients, employs the criterion “confined to bed (>72 h)” as a positive risk factor for DVT. The Padua Prediction Score, a guideline for VTE risk factors among medical inpatients, includes “reduced mobility” as a positive risk factor for VTE. The Modified Wells Clinical Score primarily relies on patient history and physical examination to preliminarily identify high-risk factors for DVT, including “recently bedridden for more than 3 days” as a positive risk factor (28, 40). Zhao et al. (41) conducted a multivariate logistic regression analysis demonstrating that immobilization (OR = 1.885, 95%CI: 1.302–2.730) and age (OR = 2.188, 95%CI: 1.354–3.537) are independent risk factors for DVT. A systematic review and meta-analysis of 43 epidemiological studies involving 24,181 VTE patients found that bed rest approximately doubles the risk of VTE. This increase may be attributed to muscle and diaphragm dysfunction in bedridden patients, leading to reduced venous blood flow in the legs and resulting in blood stasis. Furthermore, venous stasis may activate the extrinsic coagulation pathway due to hypoxemia, potentially causing endothelial damage or reducing fibrinolytic activity, thereby inducing a hypercoagulable state (42). Two retrospective studies conducted in China and one in Saudi Arabia indicate that immobilization is the most prevalent risk factor for DVT (21, 43, 44). A multicenter, prospective case–control study demonstrated that bed rest increases the incidence of DVT by a factor of 5.64 (OR = 5.64, 95% CI: 2.04–15.56, *p* = 0.008) (45). Currently, there is significant controversy surrounding the duration of bed rest and the timing of early mobilization during the acute phase of AIS patients, highlighting the need for additional research to address and clarify this issue.

Medium-frequency electrotherapy is commonly used as a treatment method for patients with acute ischemic stroke, particularly those who are severely paralyzed and bedridden. This therapy can enhance neural function and promote neural plasticity through electrical stimulation, stimulating muscle contraction to maintain muscle activity, promoting the recovery of lower limb motor function, and reducing bed rest time. Unexpectedly, this study found that medium-frequency electrotherapy may have a role in preventing deep vein thrombosis in AIS patients. Medium-frequency electrotherapy involves the application of pulsed currents at frequencies ranging from 1,000 to 100,000 Hz for muscle treatment, including Interferential Current (IFC), modulated medium-frequency electrotherapy, and symmetrical sine wave medium-frequency electrotherapy. In this study, patients received treatment on their lower limbs using a medium-frequency electrotherapy device, with electrode pads positioned on the thigh and calf muscle groups for stimulation, lasting 30 min each day. In this study, we used medium-frequency electrotherapy devices to treat the patients’ lower limbs, placing electrode pads on the thigh and calf muscle groups for stimulation, with a stimulation intensity of 2,000–8,000 Hz, once a day, each session lasting 30 min. Previous studies have demonstrated that low-frequency electrotherapy has a significant preventive effect on DVT. Low-frequency electrotherapy is a technique that employs currents with frequencies ranging from 0 to 1,000 Hz to treat various conditions, including neuromuscular electrical stimulation (NMES), functional electrical stimulation, and transcutaneous electrical nerve stimulation. A review study indicated that NMES effectively reduces the risk of lower limb DVT compared to the absence of preventive measures (OR = 0.40, 95% CI: 0.23–0.70, *p* = 0.02), providing moderate-quality evidence and is regarded as an effective mechanical approach for preventing leg thrombosis (46). Broderick et al. (47) demonstrated that NMES is more effective than intermittent pneumatic compression (IPC) in enhancing lower limb hemodynamics. However, research on the prevention of DVT using medium-frequency electrotherapy remains limited. Rampazo et al. (48) reported that medium-frequency electrotherapy has a higher carrier frequency, is more comfortable than low-frequency pulsed current, and can penetrate deeper tissues, resulting in increased muscle torque. We hypothesize that the prevention of lower limb DVT by medium-frequency electrotherapy may involve the following mechanisms: First, medium-frequency currents, due to their higher frequency, can stimulate muscle tissue at a deeper level and promote local blood circulation, inducing contraction of both normally innervated and denervated skeletal muscles, which can prevent muscle atrophy, promote skeletal muscle activity, enhance the venous pumping function of lower limb muscles, and prevent blood stasis. Secondly, medium-frequency electrotherapy may also simulate natural activity by stimulating lower limb muscle contractions, with electrical stimulation causing dorsiflexion of the tibialis anterior, and plantarflexion contractions of the gastrocnemius and soleus muscles, simulating the ankle pump motion and improving the venous pumping function of lower limb muscles. The current evidence regarding the effectiveness of medium-frequency electrotherapy in preventing DVT in patients with AIS is insufficient to draw definitive conclusions. Future high-quality randomized controlled trials are essential to provide robust evidence.

The findings of this study are not entirely consistent with the existing literature. Previous studies have indicated that risk factors, including the NIHSS, lower limb weakness, and the Barthel Index, are independently associated with DVT. However, Li et al. (49) found that NIHSS and lower limb weakness are not associated with isolated distal deep vein thrombosis following AIS (*p* > 0.05). Balogun et al. (50) found no correlation between NIHSS scores and the Barthel Index (*p* > 0.05). Both studies utilized a specific component of the NIHSS, namely the lower limb score, and regarded an elevated lower limb NIHSS score as an independent risk factor for DVT following stroke (51, 52). These variations may contribute to the development of future predictive models. Additionally, D-dimer serves as a commonly used biochemical predictor for DVT, being a specific product of fibrin degradation. Its elevated levels indicate the activation of coagulation and fibrinolytic systems within the body. The D-dimer test is characterized by high sensitivity and low specificity in laboratory evaluations. A negative D-dimer result often rules out DVT, whereas factors such as advanced age, prolonged bed rest, surgery, infection, and malignancy can lead to elevated levels. While D-dimer can predict DVT following AIS, there exist variations in threshold values across different studies (22). Due to the lack of high-quality research on individual factors, future studies should prioritize the identification and validation of clinical risk factors and biomarkers. This is essential for enhancing patient stratification and guiding the development of effective risk prediction models.

### Strengths and limitations of the study

Our study still has some limitations. This study is a retrospective study, and we were unable to adequately collect and include more research factors, such as D-dimer. First, DVT is typically diagnosed using Doppler ultrasonography. Secondly, in China, D-dimer testing is not a mandatory examination for AIS patients. Additionally, the model development was limited to one medical center, and external validation was performed at only one other center. Therefore, we still need to develop prospective, larger scale, multi-center studies for further validation in the future.

## Conclusion

In summary, we developed a nomogram for the personalized prediction of DVT in patients with AIS, which demonstrates strong predictive performance and clinical benefits. This nomogram assists clinicians in identifying high-risk populations for DVT following AIS, facilitating early targeted interventions that effectively prevent the occurrence of DVT. This is significant for reducing the incidence of DVT in patients, shortening hospital stays, and improving prognosis.

## Data Availability

The original contributions presented in the study are included in the article/supplementary material, further inquiries can be directed to the corresponding author.
